# Computer analysis of protein functional sites projection on exon structure of genes in Metazoa

**DOI:** 10.1186/1471-2164-16-S13-S2

**Published:** 2015-12-16

**Authors:** Irina V Medvedeva, Pavel S Demenkov, Vladimir A Ivanisenko

**Affiliations:** 1 The Federal Research Center Institute of Cytology and Genetics SB RAS, Novosibirsk, Russia; 2 Novosibirsk State University, Novosibirsk, Russia

**Keywords:** ligand binding sites, exon structure, exon shuffling, exon phases, exon length, discontinuous protein sites

## Abstract

**Background:**

Study of the relationship between the structural and functional organization of proteins and their coding genes is necessary for an understanding of the evolution of molecular systems and can provide new knowledge for many applications for designing proteins with improved medical and biological properties. It is well known that the functional properties of proteins are determined by their functional sites. Functional sites are usually represented by a small number of amino acid residues that are distantly located from each other in the amino acid sequence. They are highly conserved within their functional group and vary significantly in structure between such groups. According to this facts analysis of the general properties of the structural organization of the functional sites at the protein level and, at the level of exon-intron structure of the coding gene is still an actual problem.

**Results:**

One approach to this analysis is the projection of amino acid residue positions of the functional sites along with the exon boundaries to the gene structure. In this paper, we examined the discontinuity of the functional sites in the exon-intron structure of genes and the distribution of lengths and phases of the functional site encoding exons in vertebrate genes. We have shown that the DNA fragments coding the functional sites were in the same exons, or in close exons. The observed tendency to cluster the exons that code functional sites which could be considered as the unit of protein evolution. We studied the characteristics of the structure of the exon boundaries that code, and do not code, functional sites in 11 Metazoa species. This is accompanied by a reduced frequency of intercodon gaps (phase 0) in exons encoding the amino acid residue functional site, which may be evidence of the existence of evolutionary limitations to the exon shuffling.

**Conclusions:**

These results characterize the features of the coding exon-intron structure that affect the functionality of the encoded protein and allow a better understanding of the emergence of biological diversity.

## Background

The relationship between protein structure and the coding gene structure is one of the important problems of modern biology. The major research in this area was carried out to define peculiarities in the organization of the domain structure of proteins depending on the exon-intron structure of genes [1-3]. The domain structure of proteins is determined by mutual arrangement of these domains in the tertiary and primary structures. The research in this field provides important information about the function of the protein. There are several types of domains that could be defined in proteins: structural, functional and evolutional [4]. At the same time various types of domains can intersect each other but do not match to each other exactly. The structural domain is defined as an isolated unit in the space that is capable of self-assembly into the native structure, having relatively little contact with other parts of the protein and its own hydrophobic core. The functional domain can be defined as a minimal part of the polypeptide chain that is capable of self-assembly into the native structure and has the same functionality as in the structure of the complete protein [4]. The evolutionary domain is considered to be the evolutionary unit in domain shuffling. In this case, the domain is defined as the extended part of the polypeptide chain evolving significantly slower than other protein sequences. In 1981, Go introduced the term "module" [5,6], defining the structural unit that has a diameter of 15-35 Å. This structure also could be determined to be an evolutionary unit. There is evidence that modules can operate independently, so it was assumed that the module is the initial functional unit of the protein [7].

It has been shown that the boundaries of functional domains and modules of proteins that could also be considered as evolutionary units correlate with the exon boundaries in the coding gene sequence [8]. This is associated with the phenomenon of shuffling, one the major factors in protein evolution. Exon shuffling as another mechanism of gene evolution and was proposed by W. Gilbert in 1978 [9].

The events of fusion and fission of exons are also considered to be types of exon shuffling, as well as their duplication. The exon-intron structure of genes also can be characterized by a phase. The phase is defined as the break of codon by intron: 0 - if the break is between the triplets, 1 - in the case that the shift of the break is for one nucleotide, 2 - in the case that the shift of the break is for two nucleotides. So one exon has two phases at the 5'-end and 3'-end. If these phases are equal, the exon is considered symmetrical. Symmetry can be a sign of exon shuffling. The events of exon shuffling are distributed among genomes of multicellular organisms, especially in genes coding extracellular proteins and receptors. It has thus been hypothesized that this mechanism of evolution contributed to the development of multicellular phenomena [10].

It should be noted that despite the diversity of the criteria by which protein domains are defined, their common property is the ability to perform their functions, which are realized by the functional protein sites. Among the various types of functional sites, the following can be identified: the active sites that catalyse chemical reactions; ligand binding sites; allosteric sites which could alter the conformation of the protein by ligand binding; regulators which can regulate the enzymatic activity of proteins by activators and inhibitors; and sites of posttranslational modification of proteins (sites of phosphorylation, methylation, acetylation, glycosylation, addition of a fatty acid and others). Functional sites possess compactness in their spatial structure, i.e. their amino acid residues are close to each other in spatial structure but are distantly located in sequence. The mutations leading to the replacement of amino acid residues in a functional site usually are not fixed [11]. The functional sites of proteins, especially catalytic sites, are highly conservative in that the amino acid composition and the mutual spatial orientation of the components of the functional site residues are maintained in homologous proteins [12].

Currently, the majority of articles about the study and prediction of protein functional sites propose methods of searching the conservative fragments in the protein sequence and structure. Several databases of protein functional sites have been created including: SitesBase [13], PDBSite [14], eF-Site [15], sc-PDB [16], FireDB [17], and LigASite [18].

These databases are devoted to the structural organization of the various functional sites in the primary and spatial structure of proteins. However, the analysis of the general peculiarities of structural organization of the various functional sites in the exon-intron structure of the gene is still poorly understood. One of the approaches to this analysis is to match the positions of the amino acid residues of the functional sites with the boundaries of the coding exon structure of gene.

Over the past few years, many databases have been created containing integrated information such as marking the borders of exons and domains on nucleotide and amino acid sequences and the spatial structure of the protein. Such resources include: XdomView [19], ExDom [20] and the Structural Exon Database (SEDB) [21]. We have previously developed a computer system, SitEx, designed for the analysis of the relationship between the exon-intron structure of genes and the characteristics of the structural and functional organization of proteins, including the structure and properties of their domains and functional sites.

In this paper, we analysed the distribution of functional sites on the exon-intron structure of the encoding gene using the SitEx database [22]. We considered as functional sites the ligand binding sites. It was shown that the functional sites are often encoded by exons that have a more ancient origin. Further, the exons coding at least part of the functional site undergo shuffling more often. In addition, we investigated the relationship between the length and the frequency of the exons coding functional sites. We demonstrated that the length of such exons is significantly longer than the length of the exons non-coding functional sites on average, and the frequency of occurrence of introns in the sequence area of the functional site is lower than expected.

Thus, it can be assumed that the characteristics of the distribution of functional sites on the exon structure may be one of the factors for evolutionary selection, and functional sites can be seen as an evolutionary unit also.

## Results

### Analysis of the length distribution of exons coding and non-coding functional sites

**Figure 1:**
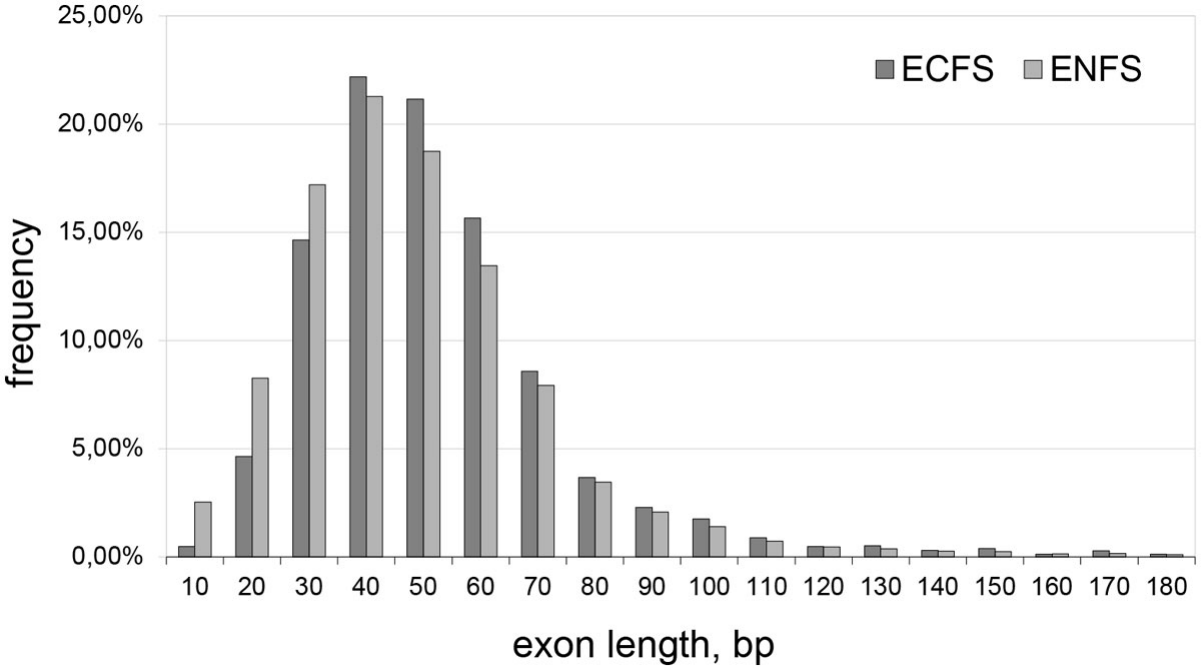
**Exon length distribution in ECFS and ENFS**.

We used samples of exons coding and non-coding functional sites (EC and ENC, respectively) using the database SitEx for a comparative analysis of the exon length distribution (Figure 1). Statistical analysis reveal a significant difference in length distribution between ECFS and ENFS using the χ2 (χ2 = 582.8, *df *= 152, *P *≈10^−16^). The average length of exons from the EC sample exceeded the average length of exons from the ENC sample. The average length of exons reached ≈159 bp and ≈144 bp, respectively (median of 129 bp and 114 bp, respectively). According to the Mann-Whitney test, the average values of the lengths of these exons differ with a significance *P *≈ 10^−6^. Thus, the average length of the EC significantly exceeds the average length of the ENC. This could be helpful to maintaining the conservative sequence area around the binding sites of amino acids that shape the spatial structure of the functional unit.

### Analysis of the ligand binding sites discontinuity

**Figure 2:**
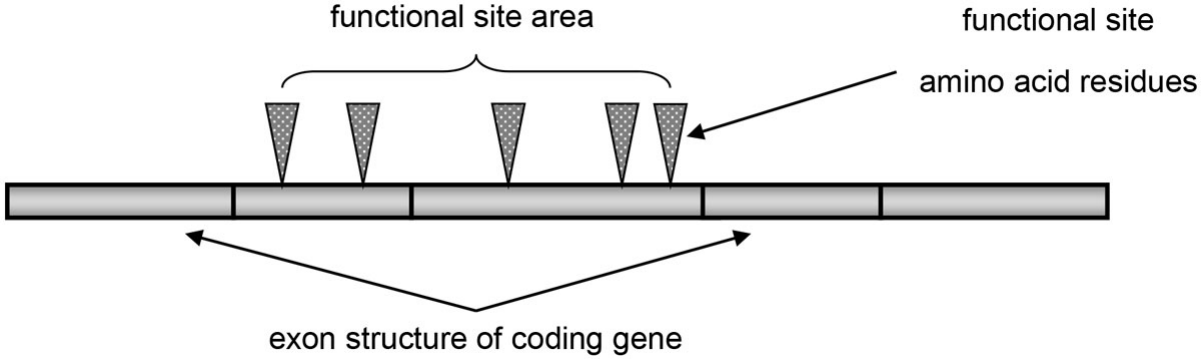
**Functional site area projected on exon structure of encoding gene**.

It is possible to estimate the functional sites discontinuity through the protein sequence and the exon structure of the encoding gene. The discontinuity of the codon encoding amino acid residue of the functional sites on the exon structure characterizes how the ECFS are distributed in sequence. Based on the fact that the functional sites tend to be encoded by longer exons, it can be assumed that the functional sites discontinuity in the exon structure is significantly less than could be expected by chance. To test this hypothesis, we estimated the expected and observed frequency of exon boundaries in the functional site area while mapping them to the protein sequences. In this case, we considered the functional site area as the protein sequence fragment that is limited by the first and last functional site amino acid positions in the protein sequence (Figure 2).

The expected distribution of the number of exon borders in the functional site area was calculated using a tenfold iteration of randomly selected positions of exon boundaries in the gene sequence. We iterated the positions of the real number of exon borders for each gene from the sample. The distributions of the observed and expected number of exons were compared by χ2 criterion (χ2 = 22.4, *P *<0.01, df = 6).

It was shown that the observed number of exons coding fragments of the amino acid sequence corresponding to the functional site area was significantly lower than the number of exons expected by random (statistical significance Mann-Whitney test U = 52988.5; N_1 _= 427; N_2 _= 390; *P *<< 0.01). A similar analysis was conducted for the different groups of the functional sites (binding sites of amines, alcohols, organic acids derivatives and complex organic compounds) widely represented in the SitEx database. In all cases, the observed number of exons coding the functional site area has been significantly lower than expected (Additional file 3).

Based on this analysis one can conclude that the functional sites of proteins are statistically more likely to be encoded by only one or adjacent exons. Thus, the amino acid residues of the functional sites are clustered, forming a structural and evolutionary unit of protein.

### Frequency of phases of exons coding functional site amino acids on the boundaries

We have analysed the occurrence of different phases of exons at the 5- 'or 3'-terminus, depending on the sequence fragments coding the functional sites of the protein. The sample included 1801 non-homologous human gene sequences from the previous sample. These sequences possess 7735 exons coding at least part of the protein functional site. The control sample contains 7281 exons that do not code the proteins functional sites and are located outside the functional sites area. While preparing the samples, the first and last exons in the coding sequence were excluded. Comparison of these samples by phase representation using contingency tables 2*2 showed significant superiority of phase 0 on the 5'- or 3'-terminus, including exons from the EC sample (5': χ2 = 8.3, *P *≈0.004; 3': χ2 = 4.7, *P *≈0.03). In the case of symmetric exons, the observed prevalence of phases 0-0 is retained (χ2 = 13.6, *P *≈2·10^−4^).

One could suggest that functional sites encoded on the exon borders impose restrictions for splicing so it was interesting to estimate the representation of intron phases that break the codon coding amino acid residue of the protein functional site. In the EC sample, there was 1801 such codons, where 52% were in phase 0 on the 5'- or 3'- exon boundary. A sample of random sequences was built using a permutation test. The positions of the functional sites of the amino acid residues were randomly changed in sequence. This rearrangement was performed 1,000 times for each sequence from the sample.

We compared phase fractions of the introns that break the codon encoding amino acid residue of the protein functional site between those observed in the EC sample and those expected from the sample built by the transposition procedure described above. The difference between fractions was shown using a χ2 contingency table (*P *≈2, 2·10^−13^, taking into account the Bonferroni correction). The percentage of introns in phase 0 in the calculated sample was 0, 64%, which is more than was in observed sample.

**Figure 3:**
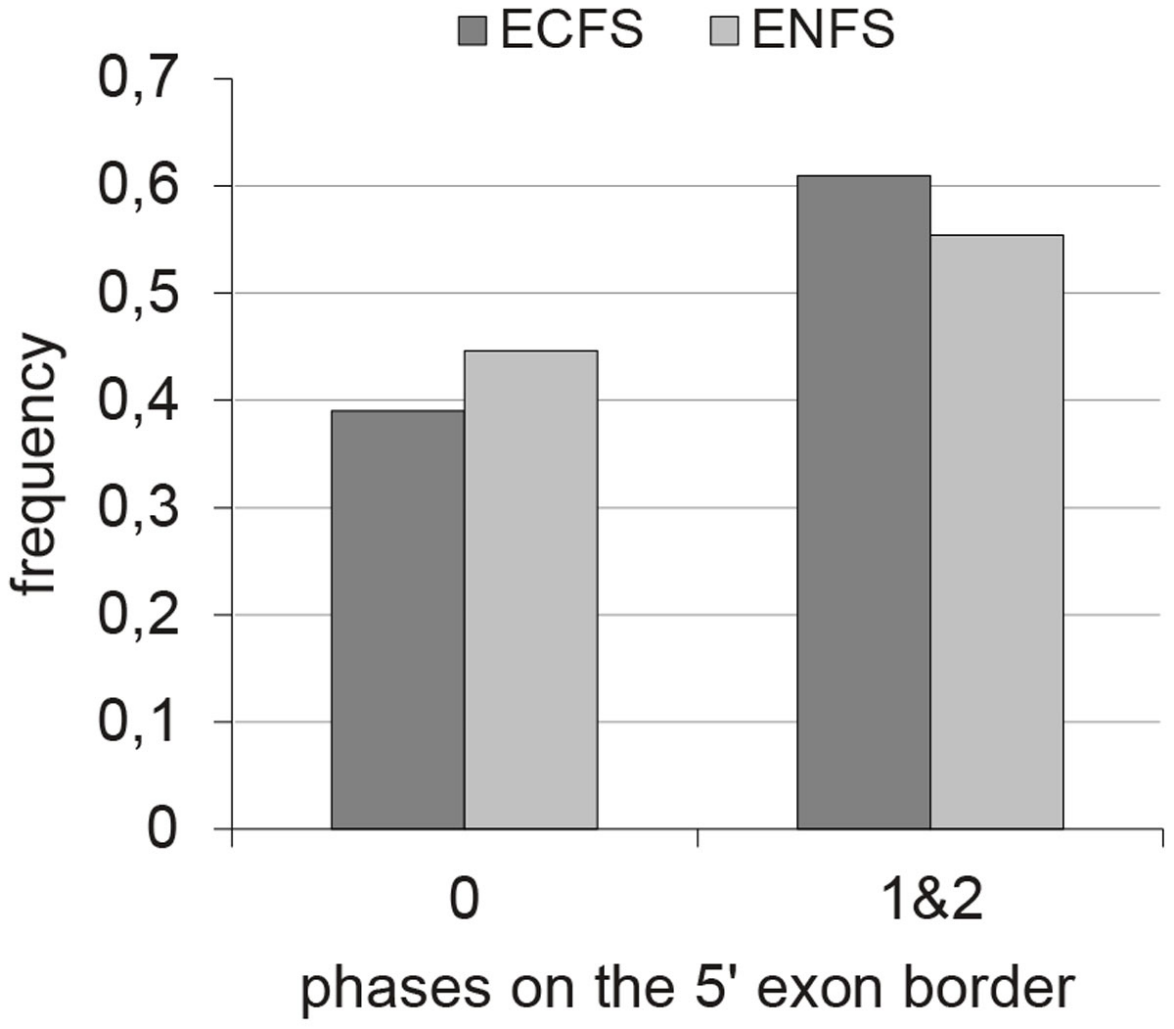
**The distribution among ECFS encoding functional site amino acid residue on 5' border and ENFS by exon phase on 5' exon border**.

Comparison of the phase 0 occurrence on the 5' exon border between the ENC and EC coding the functional site amino acid residues at the 5' exon border with a paired Wilcoxon test showed a statistically significant difference between phase 0 occurrence and the occurrence of phases 1 and 2 (*P *≈8.3 * 10^−6 ^using Bonferroni (Z = 4.86)). The mean and median for phase 0 occurrence were less (Figure 3).

## Discussion

Protein sequence variability is influenced by many factors: the rate of protein synthesis and spatial structure and protein interactions with other proteins [23]. There are also the evolutionary factors that influence at the level of the coding gene structure: exon duplication, exon shuffling, exonization of intronic sequences and mobile elements, evolution of alternative splicing, usage of alternative translation initiation and termination sites, among others. These changes affect the conserved regions of the protein that form the functional and binding sites.

We have shown that the protein functional sites tend to be coded by one or adjacent exons in the gene sequence. The discontinuity of the functional sites in the exons is significantly lower than expected randomly, and the length of the exon coding functional sites on average are significantly greater than the length of the exon non-coding functional sites.

The exon lengths have been actively studied in the literature when analysing the relationship of the exon-intron structure and alternative splicing, and the level of expression and GC-composition. In particular, it has been shown that alternatively spliced exons tended to be shorter [24,25], and where the exons are shorter, they are included more frequently in alternative forms [26]. It is also shown that the ancestors of vertebrates had longer constitutive exons than humans, whose length matched with the size of nucleosomes [27].

Further, the inverse correlation between the length of the exons in the sequence and the speed of expression in different organisms was demonstrated [27,28], as well as the complex correlation between exon length and GC-composition [29].

The performing of site functions is ensured by a spatial structure that is determined by the spatial structure of the entire protein domain. Consequently, the amino acid sequences encoded by short exons are less likely to form a stable structure and maintain site functionality. This is even more important in the case of functional sites that are of a discontinued trough exon structure of the coding gene because such sites are formed by distant amino acid residues. However, the site discontinuity in the published research was mentioned but has not been studied [30,31]. In the case of the exon shuffling event, only part of the discontinued functional site would be transferred. Thus, the functional site would not be able to operate fully, and at the same time it could disrupt the structure of other functional sites of the protein. So exon shuffling not only contributes to functional diversity, but could also break it. One can expect that the longer exons are more likely to retain their functionality after shuffling so they are important for function and maintained in evolution. The tandem duplication of exons could be an example [32]. If the exons coding ligand binding sites are adjacent they are more likely to shuffle entirely, as in the case shuffles domains. It was shown that the dominant symmetric phase for domain shuffling is 1-1 which could indicate the later origin of domain shuffling [33]. Significant predominance of phase symmetric exons among the ECFS was not found. It has previously been shown that the phase symmetric exons often code unstructured regions of proteins [34].

However, among symmetrical exons from the ECFS sample, the predominant phase is 0-0. Previously, it was shown that the phase symmetrical 0 phase is common for exons with more ancient origins, due to the phenomenon of exon shuffling as one of the main ways for the emergence of proteins with new functions [35,36]. Phases 1 and 2 are more common among the exons which appeared later. Based on our data one could suggest that there are limitations to shuffling of exons that code functional protein sites.

**Figure 4:**
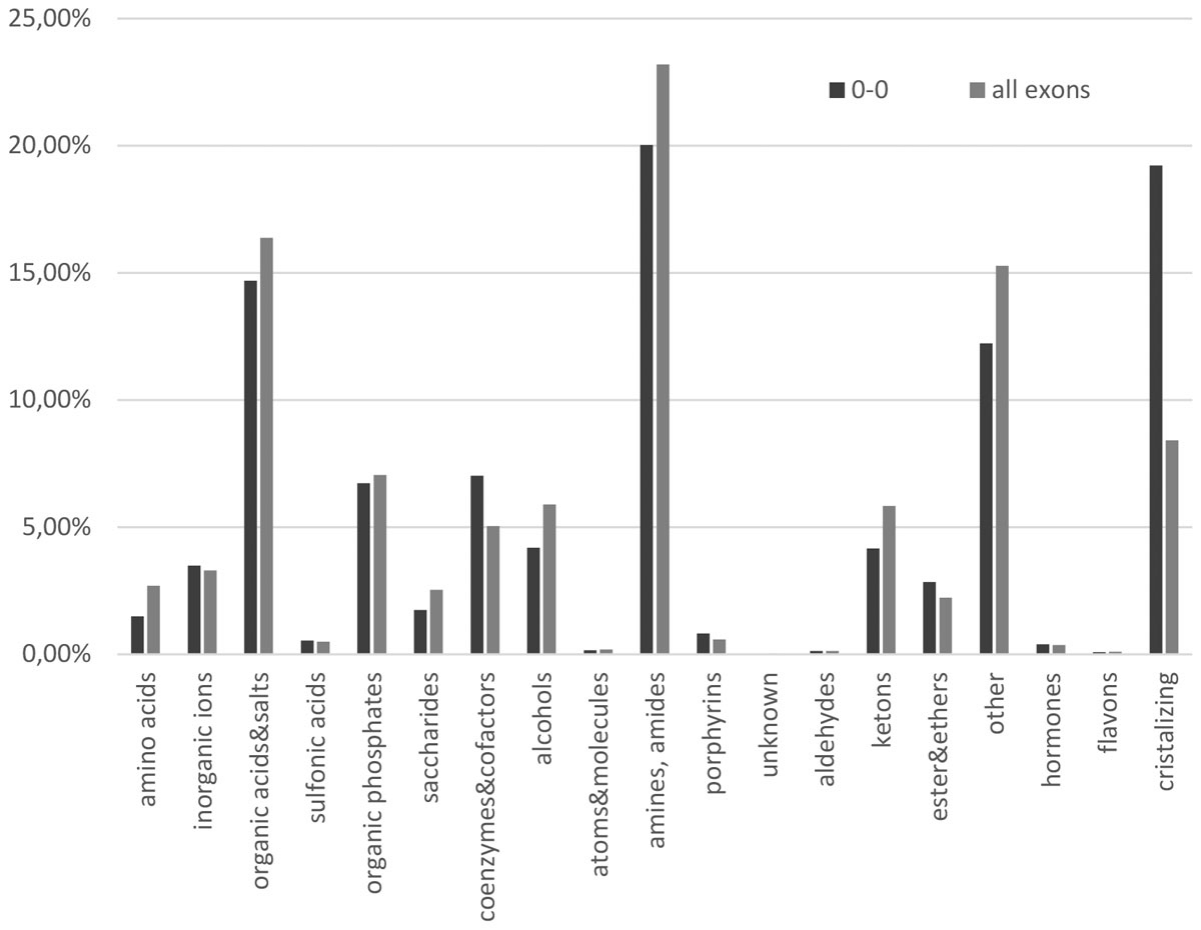
**Functional site type distribution in symmetric exons and all**.

To determine the functional site types that are encoded by symmetric exons in phase 0, we classified the ligands binding by them according to the nomenclature (Figure 4). Thus there is an increased presence of the following ligands: ethers, porphyrins, cofactors and coenzymes, and compounds, involved in the preparation for obtaining the crystal structure of proteins (solvents, precipitators, buffers etc.). Synthetic substitutes of ATP and GTP are widely represented among the esters. These ligands indicate the catalytic sites and must be encoded by the exons, which are compact enough to have been shuffled since ancient times.

It is well known that many ligands presented in 3D protein structures were added during processes of buffering and crystallization. Given this fact we applied the additional analysis for the sample that was lack such ligands (Additional file 1). It was found that the features defined for the full sample did not differ from the cut sample.

## Conclusions

Our data describe the peculiarities of the exon-intron structure, which affect the functionality of the encoded protein. We have established that protein functional sites are encoded mostly by longer exons. In the case of discontinuous functional sites, fragments of DNA encoding them are predominantly distributed within one or more contiguous exons in the gene sequence. We revealed statistically significant differences between the frequencies of the codon phases located at the ends of the exons coding and non-coding functional sites of proteins. We assume that the occurrence of functional sites in the amino acid sequences of the proteins may limit the variability of the exon structure of the gene, including exon shuffling in evolution. In the future, this issue could be clarified using homologous gene sequences.

## Methods

To evaluate the effect of protein functional sites on the encoding gene structure, we used the following data contained in SitEx database: protein sequences and the positions of the amino acid residues of the functional sites in them, gene sequences and positions of exon-intron boundaries in the corresponding gene. The information presented in SitEx about protein functional sites was retrieved from PDB entries [22]. The positions of protein functional sites were considered as long as the information about ligands for ligand binding sites. To consider the possible variability of functional sites between different types of organisms in our statistical analysis, only the Metazoa genes were examined. For the extracted sequences we applied the program SiLiX [36] to discard the similar protein sequences. 2236 different groups of proteins were selected using maximum 35% sequence similarity with minimum 80% of sequences overlap within groups. Median number of sequences per each group was 1 (see Supplementary 1 Figure 1). In the case there were more than one sequence in group we selected one sequence randomly. List of sequences included in the following analysis is presented in Additional file 2. We divided the exons from these sequences into two samples: the one where exon codes at least one protein site amino acid (sample EC: 8923 exons) and the other where exon do not code any protein site amino acid (sample ENC: 15145 exons).

There are about 7 chains presented in PDB structures for each protein in our sample (see Supplementary 1 Figure 2). We collected the information about 46659 protein ligand binding sites, that could overlap between structures of one protein. There were 6 sites and 15 functional sites amino acids per protein in median in our sample (see Supplementary 1 Figure 3 and 4). Many sites bind the same ligand in different proteins, so the number of unique ligands was 3659. All ligands could be divided into 18 groups including derivatives of amino acids, amines, organic acids and etc. (Figure 4). Also we defined the group of ligands that are more likely to be involved in buffering and protein crystallization process (Supplementary Table 1).

Using created sample we divided the exons into two groups for the following:

1. exons that code functional sites of amino acids (EC);

2. exons that do not code functional sites of amino acids (ENC);

We applied R environment for statistical analysis and Perl for organizing data.

## List of abbreviations used

ECFS - exons that code functional sites of amino acids

ENFS - exons that do not code functional sites of amino acids

## Competing interests

The authors declare that they have no competing interests.

## Authors' contributions

The computer experiments were designed by IM, PD and VI. IM and PD performed statistical analysis. VI and IM wrote the manuscript. VI supervised the research. All authors read and approved the manuscript.

## Supplementary Material

Additional file 3 Click here for file

Additional file 1 Sample characteristics Click here for file

Additional file 2 List of protein sequences included in the analysis. Click here for file

## Declarations

Publication of this article has been funded by Russian Science Foundation.

This article has been published as part of *BMC Genomics *Volume 16 Supplement 13, 2015: Selected articles from the 7th International Young Scientists School "Systems Biology and Bioinformatics" (SBB'2015): Genomics. The full contents of the supplement are available online at http://www.biomedcentral.com/bmcgenomics/supplements/16/S13.

## References

[B1] Liu M Walch H Wu Sh Grigoriev A Significant expansion of exon-bordering protein domains during animal proteome evolution Nucl Acids Res 2005 33 1 95 105 15640447 10.1093/nar/gki152 PMC546140

[B2] Kawashima T Kawashima S Tanaka C Domain shuffling and the evolution of vertebrates Genome Research 2009 19 8 1393 1403 19443856 10.1101/gr.087072.108 PMC2720177

[B3] Cancherini DV França GS de Souza SJ The role of exon shuffling in shaping protein-protein interaction networks BMC Genomics 2010 11 Suppl 5 S11 21210967 10.1186/1471-2164-11-S5-S11 PMC3045794

[B4] Ponting CP Russell RR The natural history of protein domains Annu Rev Biophys Biomol Struct 2002 31 45 71 11988462 10.1146/annurev.biophys.31.082901.134314

[B5] Takahashi K Noguti T Hojo H Yamauchi K Kinoshita M Aimoto S Ohkubo T Go M A mini-protein designed by removing a module from barnase: molecular modeling and NMR measurements of the conformation Protein Eng 1999 12 8 673 680 10469828 10.1093/protein/12.8.673

[B6] Go M Correlation of DNA exonic regions with protein structural units in haemoglobin Nature 1981 291 5810 90 92 7231530 10.1038/291090a0

[B7] Yanagawa H Yoshida K Torigoe C Park JS Sato K Shirai T Go M Protein anatomy: functional roles of barnase module J Biol Chem 1993 268 8 5861 5865 7680649

[B8] Kaessmann H Zöllner S Nekrutenko A Li W-H Signatures of Domain Shuffling in the Human Genome Genome Research 2002 12 11 1642 1650 12421750 10.1101/gr.520702 PMC187552

[B9] Gilbert W Why genes in pieces? Nature 1978 271 5645 501 622185 10.1038/271501a0

[B10] França GS Cancherini DV De Souza SJ Evolutionary history of exon shuffling Genetics 2012 140 4-6 249 257 10.1007/s10709-012-9676-3 22948334

[B11] Binkowski TA Cuff M Nocek B Chang C Joachimiak A Assisted assignment of ligands corresponding to unknown electron density Journal of structural and functional genomics 2010 11 1 21 30 20091237 10.1007/s10969-010-9078-7 PMC2885970

[B12] Todd AE Orengo CA Thornton JM Evolution of function in protein superfamilies, from a structural perspective J Mol Biol 2001 307 4 1113 43 11286560 10.1006/jmbi.2001.4513

[B13] Gold ND Jackson RM SitesBase: a database for structure-based protein-ligand binding site comparisons Nucleic Acids Research 2006 34 Database issue D231 D234 16381853 10.1093/nar/gkj062 PMC1347425

[B14] Ivanisenko VA Pintus SS Grigorovich DA Kolchanov NA PDBSite: a database of the 3D structure of protein functional sites Nucleic Acids Research 2005 33 Database issue D183 D187 15608173 10.1093/nar/gki105 PMC540059

[B15] Kinoshita K Furui J Nakamura H Identification of protein functions from a molecular surface database, eF-site J Struct Func Genomics 2002 2 1 9 22 10.1023/a:1011318527094 12836670

[B16] Kellenberger E Muller P Schalon C Bret G Foata N Rognan D sc-PDB: an annotated database of druggable binding sites from the Protein Data Bank J Chem Inf Model 2006 46 2 717 727 16563002 10.1021/ci050372x

[B17] Lopez G Valencia A Tress M FireDB-a database of functionally important residues from proteins of known structure Nucleic Acids Res 2007 35 Database issue D219 D223 17132832 10.1093/nar/gkl897 PMC1716728

[B18] Dessailly B Lensink M Orengo C Wodak S LigASite: a database of biologically relevant binding sites in proteins with known apo-structures Nucleic Acids Res 2008 36 Database issue D667 D673 17933762 10.1093/nar/gkm839 PMC2238865

[B19] Vivek G Tan TW Ranganathan S XdomView: protein domain and exon position visualization Bioinformatics 2003 19 1 159 160 12499310 10.1093/bioinformatics/19.1.159

[B20] Bhasi A Philip P Manikandan V Senapathy P ExDom: an integrated database for comparative analysis of the exon-intron structures of protein domains in eukaryotes Nucleic Acids Res 2009 37 suppl 1 D703 D711 18984624 10.1093/nar/gkn746 PMC2686582

[B21] Leslin CM Abyzov A Ilyin VA Structural exon database, SEDB, mapping exon boundaries on multiple protein structures Bioinformatics 2004 20 11 1801 1803 14988102 10.1093/bioinformatics/bth150

[B22] Medvedeva I Demenkov P Kolchanov N Ivanisenko V SitEx: a computer system for analysis of projections of protein functional sites on eukaryotic genes Nucleic Acids Research 2012 40 Database issue D278 D283 22139920 10.1093/nar/gkr1187 PMC3245165

[B23] Teichmann SA Grishin N Sequences and Topology: Evolution of proteins and evolution of computational approaches Current opinion in structural biology 2010 20 3 333 334 20483587 10.1016/j.sbi.2010.04.004 PMC4504200

[B24] Koralewski TE Krutovsky KV Valcarcel J Evolution of Exon-Intron Structure and Alternative Splicing PLoS ONE 2011 6 3 e18055 21464961 10.1371/journal.pone.0018055 PMC3064661

[B25] Gelfman S Burstein D Penn O Changes in exon-intron structure during vertebrate evolution affect the splicing pattern of exons Genome Research 2012 22 1 35 50 21974994 10.1101/gr.119834.110 PMC3246205

[B26] Roy M Kim N Xing Y Lee C The effect of intron length on exon creation ratios during the evolution of mammalian genomes RNA 2008 14 11 2261 2273 18796579 10.1261/rna.1024908 PMC2578852

[B27] Carmel L Koonin EV A Universal Nonmonotonic Relationship between Gene Compactness and Expression Levels in Multicellular Eukaryotes Genome Biology and Evolution 2009 1 382 390 20333206 10.1093/gbe/evp038 PMC2817431

[B28] Pingault L Choulet F Alberti A Deep transcriptome sequencing provides new insights into the structural and functional organization of the wheat genome Genome Biology 2015 16 1 29 25853487 10.1186/s13059-015-0601-9 PMC4355351

[B29] Zhu L Zhang Y Zhang W Yang S Chen J-Q Tian D Patterns of exon-intron architecture variation of genes in eukaryotic genomes BMC Genomics 2009 10 47 19166620 10.1186/1471-2164-10-47 PMC2636830

[B30] Wells JA McClendon CL Reaching for high-hanging fruit in drug discovery at protein-protein interfaces Nature 2007 450 7172 1001 1009 18075579 10.1038/nature06526

[B31] Liu T Altman RB Beard DA Using Multiple Microenvironments to Find Similar Ligand-Binding Sites: Application to Kinase Inhibitor Binding PLoS Computational Biology 2011 7 12 e1002326 22219723 10.1371/journal.pcbi.1002326 PMC3248393

[B32] Letunic I Copley RR Bork P Common exon duplication in animals and its role in alternative splicing Hum Mol Genet 2002 11 13 1561 1567 12045209 10.1093/hmg/11.13.1561

[B33] Kaessmann H Zöllner S Nekrutenko A Li W-H Signatures of Domain Shuffling in the Human Genome Genome Research 2002 12 11 1642 1650 12421750 10.1101/gr.520702 PMC187552

[B34] Schad E Kalmar L Tompa P Exon-phase symmetry and intrinsic structural disorder promote modular evolution in the human genome Nucleic Acids Research 2013 41 8 4409 4422 23460204 10.1093/nar/gkt110 PMC3632108

[B35] Vibranovski MD Sakabe NJ de Oliveira RS de Souza SJ Signs of Ancient and Modern Exon-Shuffling Are Correlated to the Distribution of Ancient and Modern Domains Along Proteins J Mol Evol 2005 61 3 341 350 16034650 10.1007/s00239-004-0318-y

[B36] Patthy L Genome evolution and the evolution of exon-shuffling-a review Gene 1999 238 1 103 114 10570989 10.1016/s0378-1119(99)00228-0

[B37] Miele V Penel S Duret L Ultra-fast sequence clustering from similarity networks with SiLiX BMC Bioinformatics 2011 12 116 21513511 10.1186/1471-2105-12-116 PMC3095554

