# Supplementary material for: Computer analysis of protein functional sites projection on exon structure of genes in Metazoa
Source: BMC Genomics. 2015 Dec 16;16(Suppl 13):S2. doi: 10.1186/1471-2164-16-S13-S2 (PMC4686782; doi:10.1186/1471-2164-16-S13-S2)
Supplement: Additional file 3 [file 1471-2164-16-S13-S2-S3.pdf]

Supplementary 3

Table 1. Results of Mann-Whitney test for main ligand groups presented in sample for testing hypothesis of protein functional sites discontinuity.

Ligand type	Mann-Whitney test
amino acids and its derivatives	U=24, N1=21, N2=15, p<0.01
organic acids and its derivatives	U=85.5, N1=23, N2=17, p=0.002
amines and its derivatives	U=86, N1=27, N2=16, p=0.0008
alcohols and its derivatives	U=32.5, N1=27, N2=13, p<0.01
complex organic compounds	U=43.5, N1=19, N2=19, p<0.01
