# Supplementary material for: Computer analysis of protein functional sites projection on exon structure of genes in Metazoa
Source: BMC Genomics. 2015 Dec 16;16(Suppl 13):S2. doi: 10.1186/1471-2164-16-S13-S2 (PMC4686782; doi:10.1186/1471-2164-16-S13-S2)
Supplement: Additional file 1 — Sample characteristics [file 1471-2164-16-S13-S2-S1.pdf]

Supplementary 1. Sample characteristics.

Figure 1. Distribution of number of proteins in groups formed using SiLiX in the sample.

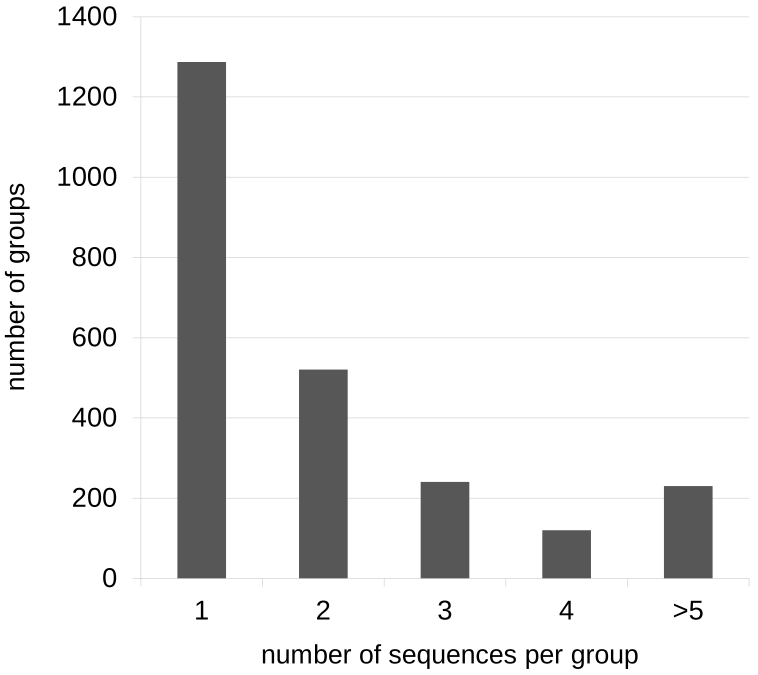

Figure 2. Distribution of number of protein chains per protein from the sample

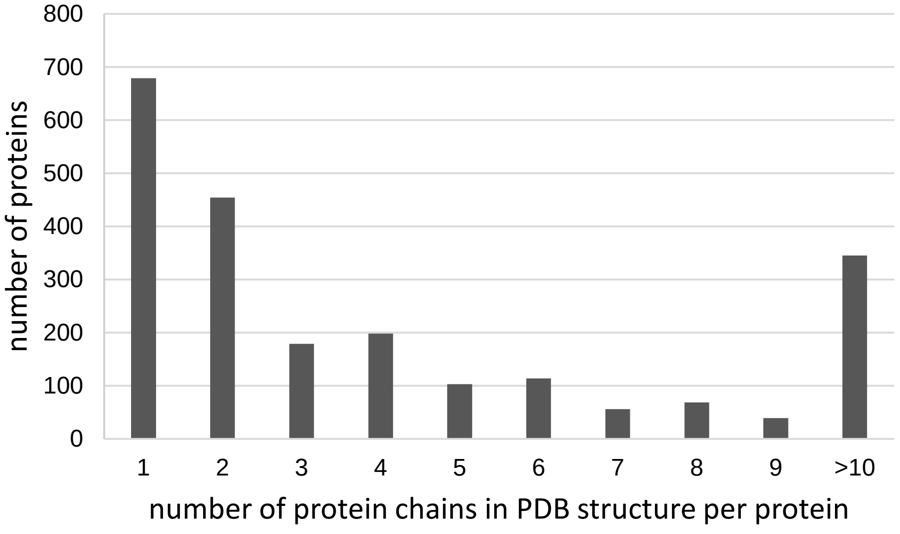

Figure 3. Distribution of number of sites per protein including sites from all protein chains in PDB.

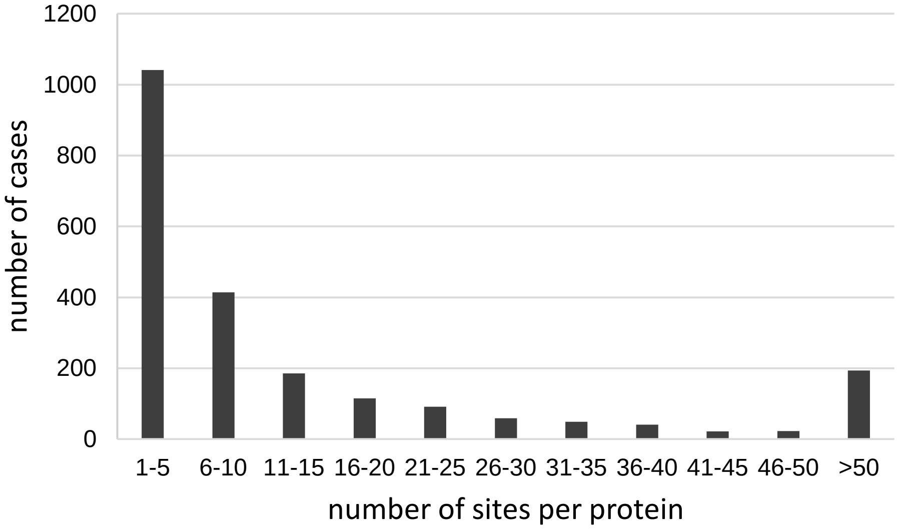

Table 1. List of ligands more likely to be involved in buffering and protein crystallization.

```
HG MERCURY (II) ION
CD CADMIUM ION
PB LEAD (II) ION
AG SILVER ION
EOH ETHANOL
MOH METHANOL
EPE 4-(2-HYDROXYETHYL)-1-PIPERAZINEETHANESULFONIC ACID
PIN PIPERAZINE-N,N'-BIS(2-ETHANESULFONIC ACID)
BCN BICINE
TRS 2-AMINO-2-HYDROXYMETHYL-PROPANE-1,3-DIOL
15P POLYETHYLENE GLYCOL (N=34)
SO4 SULFATE ION
PO4 PHOSPHATE ION
NH4 AMMONIUM ION
IPA ISOPROPYL ALCOHOL
MES 2-(N-MORPHOLINO)-ETHANESULFONIC ACID
MPD (4S)-2-METHYL-2,4-PENTANEDIOL
ACT ACETATE ION
FLC CITRATE ANION
IMD IMIDAZOLE
EDO 1,2-ETHANEDIOL
GOL GLYCEROL
CA CALCIUM ION
NA SODIUM ION
CO COBALT (II) ION
CL CHLORIDE ION
```
